# Kidney transplantation outcomes in patients with IgA nephropathy and other glomerular and non-glomerular primary diseases in the new era of immunosuppression

**DOI:** 10.1371/journal.pone.0253337

**Published:** 2021-08-17

**Authors:** Sophia Lionaki, Ilias Makropoulos, Konstantinos Panagiotellis, George Vlachopanos, Ioannis Gavalas, Smaragdi Marinaki, George Liapis, Ioannis Michelakis, Ioannis Bokos, Ioannis Boletis

**Affiliations:** 1 Department of Nephrology &Transplantation Unit, Laiko Hospital, Faculty of Medicine, National and Kapodistrian University of Athens, Athens, Greece; 2 Department of Pathology, Faculty of Medicine, National and Kapodistrian University of Athens, Athens, Greece; 3 Transplantation Unit, Laiko Hospital, Athens, Greece; Imperial College Healthcare NHS Trust, UNITED KINGDOM

## Abstract

**Objectives:**

Kidney transplant (KTx) recipients with IgAN as primary disease, were compared with recipients with other causes of renal failure, in terms of long-term outcomes.

**Methods:**

Ninety-nine KTx recipients with end-stage kidney disease (ESKD) due to IgAN, were retrospectively compared to; i/ a matched case-control group of patients with non-glomerular causes of ESKD, and ii/ four control groups with ESKD due to glomerular diseases; 44 patients with primary focal segmental glomerulosclerosis (FSGS), 19 with idiopathic membranous nephropathy (IMN), 22 with lupus nephritis (LN) and 21 with pauci-immune glomerulonephritis (PIGN).

**Results:**

At end of the observation period, graft function and survival, were similar between KTx recipients with IgAN and all other groups, but the rate of disease recurrence in the graft differed significantly across groups. The rate of IgAN recurrence in the graft was 23.2%, compared to 59.1% (p<0.0001) in the FSGS group, 42.1% (p = 0.17) in the IMN group, and 0% in the LN and PIGN groups (p = 0.01). IgAN recipients, who were maintained with a regimen containing tacrolimus, experienced recurrence less frequently, compared to those maintained with cyclosporine (p = 0.01). Graft loss attributed to recurrence was significantly higher in patients with FSGS versus all others.

**Conclusion:**

Recipients with IgAN as primary disease, experienced outcomes comparable to those of recipients with other causes of ESKD. The rate of IgAN recurrence in the graft was significantly lower than the rate of FSGS recurrence, but higher than the one recorded in recipients with LN or PIGN. Tacrolimus, as part of the KTx maintenance therapy, was associated with lower rates of IgAN recurrence in the graft, compared to the rate cyclosporine.

## Introduction

IgA nephropathy (IgAN) is the most common type of primary glomerular disease worldwide [1], with 30% of cases reaching end-stage kidney disease (ESKD) two decades after the initial diagnosis. Kidney transplantation (KTx) is the treatment of choice for these patients [2–4], but IgAN recurrence in the graft remains a threat to graft survival [5]. Pathogenesis of IgAN is related to autoantibodies directed against IgA1 with poor O-glysosylation [6]. The process of antibodies production is influenced by both B and T lymphocytes. Patients who undergo KTx are typically maintained with a triple scheme of immunosuppressants, targeting the immune system at multiples sites, in order to prevent rejection. The optimal maintenance immunosuppressive therapy for KTx is still not established, but important advances in the field have been developed during the past two decades, including the introduction of agents such as mycophenolate mofetil, tacrolimus, and mammalian target of rapamycin inhibitors for maintenance therapy, and monoclonal antibodies that bind to the α-chain of the interleukin-2 receptor for induction therapy. Previous studies in recipients with a history of IgAN have revealed contrasting results regarding graft survival, depending on the methodology and the length of observation, while the probability of disease recurrence in the graft was significantly higher in the years before 2000 [7].

The purpose of this study was to explore the long-term outcomes of KTx recipients with IgAN, during the new era of immunosuppressive agents, compared to those of recipients with other types of primary disease, glomerular or not, and estimate the frequency of disease recurrence in the graft and its impact in graft function and survival.

## Materials & methods

### Study design and definitions

**Fig 1:**
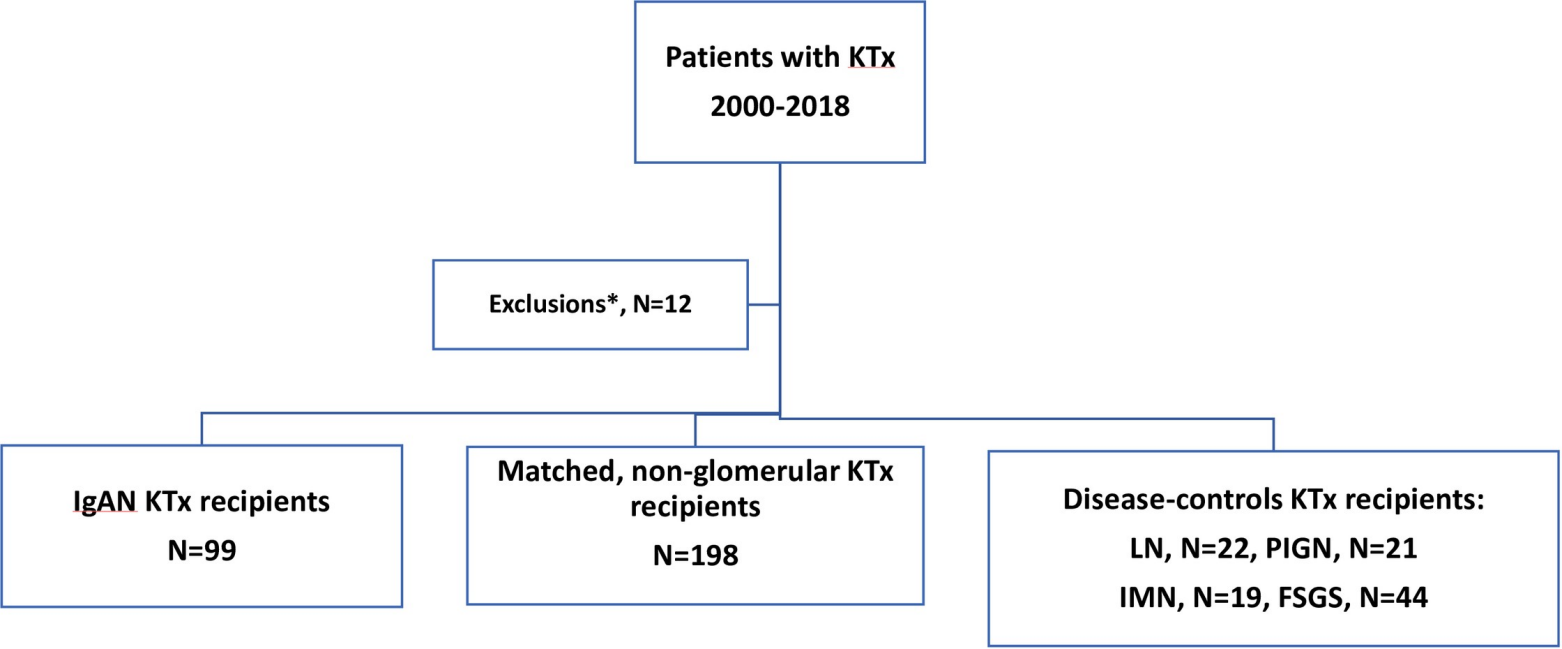
Study design.

This was a retrospective study and all data were anonymized prior to start of research. Included patients were required to have biopsy-proven IgAN in a native kidney biopsy. All of them received a kidney transplant after 2000 in the transplant center of Laiko Hospital, in Athens Greece, and had been followed up for one year or more after KTx. They were compared to i/ a case-control group, consisted of patients with non-glomerular disease causes of ESKD, including the following; polycystic kidney disease, congenital hypoplastic kidneys and obstructive uropathy. Patients and non-glomerular disease controls were matched for age, gender, donor source, and period of KTx. Matching for age permitted a maximum of five years difference between cases and controls, and a maximum of one-year difference in the date of KTx, ii/ four disease-control groups consisted of recipients with ESKD due to biopsy-proven primary focal segmental glomerulosclerosis (FSGS), after exclusion of cases with familial FSGS, idiopathic membranous nephropathy (IMN), lupus nephritis (LN) and pauci-immune glomerulonephritis (PIGN), all transplanted during the same time-period (Fig 1) in the same center.

Outcomes of interest included graft function and survival at 1^st^ post-KTx year and at the end of the observation period, as well as graft survival and mortality in long-term. The rate of IgAN recurrence in the graft was estimated and was compared to the corresponding rate in each glomerular-disease control group. Graft function was evaluated by serum creatinine measurements and glomerular filtration rate estimations, using the Modification of Diet in Renal Disease Study Equation for Estimating Glomerular Filtration Rate formula [8]. Recorded information included demographics, clinical and laboratory characteristics related to donors and recipients, i.e age, donor source, duration of dialysis prior to KTx, human leukocyte antigen mismatch, % percentage of panel reactive antibodies, and cold ischemia time. Patients were considered to be in high immunological risk if they had any of the following; ABO incompatibility, re-transplantation, panel reactive antibodies >50%. Delayed graft function was defined as the need for dialysis during the first post-transplant week. Acute rejection and recurrence of the primary disease in the graft were always confirmed by histopathology, following ultrasound-guided percutaneous biopsy. Mortality was defined as death from any cause. All cause graft loss was defined as the combination of mortality and death, censored at graft failure, leading to recommence of chronic dialysis. Non-adherence was defined as patient admission of medication non-compliance, and/or documented drug levels of the related medication below the detectable limit. Patients who had a history of non-adherence were excluded from the present study. The observation period started the day of transplant surgery and ended the day of the latest visit to the KTx clinic, or the date of death with functioning graft, or the date of initiation of chronic dialysis. Patients with a history of major surgical complication during the first post-transplant month, a history of non-compliance and those with less than one year of follow up were excluded (Fig 1).

#### Human leukocyte antigen typing and cross match methodology

Patients and donors were typed at human leukocyte antigen A, B, C, DRB1 and DQB1 loci, using commercially available serologic typing trays, as well as by low resolution molecular methods. Screening for human leukocyte antigen antibodies included both anti-human globulin complement-dependent microlymphocytotoxicity method, globulin-enhanced, complement-dependent cytotoxicity and the Luminex technique. Human leukocyte antigen sensitization of each patient was expressed as % panel reactive antibodies. Patients were required to have a negative, current, IgG anti-human globulin complement-dependent microlymphocytotoxicity method, globulin-enhanced, complement-dependent cytotoxicity cross match and T and B cell flow cytometry crossmatch, while a positive IgM complement-dependent cytotoxicity cross match was not a contraindication to KTx. The clinical and research activities being reported are consistent with the Principles of the Declaration of Istanbul as outlined in the ’Declaration of Istanbul on Organ Trafficking and Transplant Tourism’.

### Graft biopsy policy and evaluation

Graft biopsies were performed by clinical indication, i.e acute dysfunction of doubtful origin, and/or persistent proteinuria (>500 mg/day), and/or microscopic hematuria with active urine sediment i.e characterized by the presence of dysmorphic red blood cells in microscopic analysis of the urine. Histopathological evaluation was performed using formalin fixed paraffin embedded tissue sections of 3 μm thickness, stained with hematoxyline and eosin, as well as PAS, Masson and Silver histochemical stains for each biopsy specimen. A small part of cortex was appropriately treated for indirect immunofluorescence examination in frozen sections. C4d immunohistochemical stain was assessed in all tissue samples. Adequate tissue samples included a minimum of ten glomeruli and two arteries in serial sections. In light microscopy, size of glomeruli, hypercellularity, global and segmental glomerular sclerosis, adhesions with Bowman’s capsule and glomerular ischemic changes as well as tubular atrophy, interstitial fibrosis, changes in vessels walls and all features indicating rejection were recorded [9]. All specimens were examined for peritubular capillaritis, glomerulitis and transplant glomerulopathy while peritubular capillary multilaminations and other changes were appreciated by electron microscopy as indicated. Diagnosis of IgAN recurrence in the graft was determined by immunofluorescence positive staining for IgA in the mesangial area and was classified using the Oxford criteria [10].

### Immunosuppressive regimens

All patients who were transplanted after 2000 received induction treatment with an anti-interleukin 2 monoclonal antibody, while sporadic patients, who were considered to have a high likelihood of experiencing delayed graft function, received rAnti-thymocyte globulin. Maintenance therapy for KTx typically consisted of three elements; a calcineurin inhibitor, cyclosporine or tacrolimus, combined with a mycophenolate mofetil formulation and low dose methyl-prednisolone. During the first post-transplant year, patients who were maintained with cyclosporine were required to have a C2 level between 700–900 mg/dl and while those who were maintained with tacrolimus were required to have a trough level between 6–8 ng/ml, which were lowered afterwards to 500–700 mg/dl and 5–7 ng/ml respectively.

### Statistical methods

Mean values and standard deviations or median values with interquartile ranges were calculated for continuous variables and categorical variables were presented as percentages. Student t-test procedure and Mann-Whitney U test for independent samples was used to examine the potential differences between groups, regarding continuous variables of interest. Chi-square test was used to test if there was any association between groups with respect to certain categorical variables. Fischer’s exact test was used to compare categorical variables. Logistic regression was applied to identify any difference in the risk of graft failure and death between groups. Data were analyzed using Stata 13.0 software (Stata Corporation, College Station, TX) and significance was set at α = 0.05. All tests proceeded as two-tailed.

## Results

### Description of study population

Ninety-nine KTx recipients, all of Greek origin, with biopsy-proven IgAN, as cause of renal failure, were compared to 198 matched controls with non-glomerular primary diseases and four disease-control groups including, 44 patients with ESKD due to primary FSGS, 19 due to IMN, 22 due to LN and 24 due to PIGN (Fig 1). Baseline characteristics of IgAN patients and controls are displayed in Table 1. The mean age of IgAN patients at the time of KTx was 43.4 years, similar to all groups except the FSGS one (p<0.0001) (Table 1). The majority of of IgAN patients (70.7%) were males, as in all other groups except the LN controls, (p<0.0001). The mean time from IgAN diagnosis to ESKD was 5.25 (±7.6) years, alike all other groups except patients with PIGN 0.91 (±2.8) years (p = 0.0002). The mean time in dialysis prior to KTx was comparable between patients with IgAN as primary disease and all other groups. Nearly fifty nine percent of patients with IgAN (58.6%) received a graft from a living donor, similarly to all groups, with the exception of patients with IMN (p = 0.03), who were transplanted more frequently from deceased donors (Table 1).

**Table 1:** Comparison of baseline characteristics of KTx recipients with IgAN as primary disease versus all other groups of controls with primary FSGS, IMN, LN, and PIGN.

Characteristic	IgAN—KTx	Non-Glomerular Disease KTx	FSGS- KTx	IMN- KTx	LN—KTx	PIGN- KTx
Mean (±sd), median(IQR) or N (%)	Ν = 99	N = 198	Ν = 44	Ν = 19	N = 22	N = 21
Recipient’s age (years)	43.4(10.3)	42.9 (13.06)	30.9 (11.5) p<0.0001	47.1(11.5) p = 0.12	41.2(14.2) p = 0.43	43.7(13.1) p = 0.90
Gender (Μales)	70 (70.7)	140 (70.7)	13 (72.2) p = 0.89	14 (73.7) p = 0.79	4 (18.2) p<0.0001	13 (61.9) p = 0.42
Donor source (deceased)	41 (41.4)	82(41.4)	6 (33.3) p = 0.52	13 (68.1) p = 0.03	10 (45.4) p = 0.73	9 (42.85) p = 0.92
Donor’s age (years)	54.3(15.15)	53.79 (14.74)	47.5 0) p = 0.07	51.6(15.4) p = 0.47	56.05 (11.9) p = 0.61	53.4 (15.1) p = 0.80
Panel reactive antibodies >50%	5 (5.1)	12 (6.0)	0 p = 0.37	0 p = 0.61	4 (18.2) p = 0.035	3 (14.3) p = 0.1275
Disease duration prior to ESKD (years)	5.25 (7.56)	-	7.05 (4.8) p = 0.75	4.41 (4.74) p = 0.37	4.8 (5.12) p = 0.78	0.91 (2.82) p = 0.0002
Dialysis duration prior to KTx (months)	31.6 (60)	62.2 (55.8) p = 0.46	38.1(35.0) p = 0.92	38.9 (37.6) p = 0.90	45 (77) p = 0.87	34.8 (56.1) p = 0.32
Cold ischemia time	17.5(6.7)	18.5 (6.0)	16.8 (3.1) p = 0.67	17.0(5.9) p = 0.76	17.9(5.9) p = 0.79	16.9(7.1) p = 0.71
Induction therapy						
Anti-CD 25 inhibitor	96 (96.7)	178 (90.9)	17 (94.4)	17 (89.5)	21 (95.45)	20 (95.2)
Rabbit Antithymocyte globulin	3 (3.3)	7 (3.35)	1(5.6) p = 0.87	2 (10.5) p = 0.16	1 (4.5) p = 0.76	1 (4.8) p = 0.75
Maintenance immunosuppression						
MMF + CIN + GCs	85 (85.8)	189 (95.45)	18 (100)	19 (100)	21 (95.5)	20 (95.2)
MMF + mTOR + GCs	3 (3.0)	3 (1.5)	0	0	0	0
mTOR + CIN + GCs	12 (12.1)	6 (3.0)	0	0	1 (4.5)	1 (4.8)
Delayed graft function	21 (23.1)	55 (27.7)	2 (11.1)	3 (15.8)	4 (18.2)	4 (19.0)

Abbreviations: IgAN; IgA nephropathy, FSGS; focal segmental glomerulosclerosis, IMN; idiopathic membranous nephropathy, LN; lupus nephropathy, PIGN; pauci immune glomerulonephritis, ESKD; end-stage kidney disease, MMF; mycophenolate mofetil, CIN; calcineurin inhibitor, mTOR; mTOR inhibitor, GCs; glucocorticoids.

Comparisons between IgAN-KTx recipients versus all other groups of controls with glomerular disease as primary cause of ESKD is depicted by the p-value in each category.

### Kidney transplantation outcomes

#### Graft function

The mean observation period was 92.7 (±104) months for IgAN patients, and 95.7 (±110) months for patients with non-glomerular diseases (Table 2), similar to the one of patients with LN and PIGN, but longer from patients with IMN and FSGS (Table 3). Overall, long-term graft function was similar in IgAN recipients, compared to the matched non-glomerular disease controls and patients with other types of glomerular diseases. Graft function at end was superior in patients maintained with tacrolimus, compared to patients who didn’t not receive tacrolimus as part of their maintenance regimen, in both IgAN cases and the matched non-glomerular disease controls. Specifically, 68 (68.7%) patients within the IgAN group were maintained with a regimen including tacrolimus and had a mean serum creatinine of 1.5 (±0.5) mg/dl at the end of the observation period, versus a serum creatinine of 1.7 (±0.45) mg/dl in patients, who were maintained without tacrolimus (p = 0.013) within the same study-group. Accordingly, in the matched non-glomerular disease group, 153 (77.3%) patients who were maintained with a regimen containing tacrolimus had a mean serum creatinine of 1.3 (±0.5) mg/dl at end, versus a creatinine of 1.4 (±0.3) mg/dl in patients who were maintained with a regimen without tacrolimus (p = 0.04). Moreover, IgAN patients maintained with cyclosporine, mycophenolate mofetil and glucocorticoids (N = 17) had a serum creatinine of 1.9 (±1.2) mg/dl at end, while patients in the same group who were maintained with tacrolimus, mycophenolate mofetil and glucocorticoids had a serum creatinine of 1.5 (±0.0.5) mg/dl (p = 0.0345).

**Table 2:** Comparison of outcomes between KTx recipients with IgAN as primary disease and a matched control group of patients with non-glomerular causes of ESKD.

Characteristic	IgAN-KTx	Matched, non-GD KTx	p- value
Median (IQR) or N (%)	Ν = 99	Ν = 198	
Ser. creatinine 1^st^ discharge (mg/dl)	1.53 (0.55)	1.53 (0.66)	0.78
Estimated GFR at 1^st^ discharge (ml/min/1.73m^2^)	52 (19)	48 (26)	0.82
Ser. creatinine 1^st^ year (mg/dl)	1.4 (0.5)	1.4 (0.58)	0.75
Estimated GFR at 1^st^ year (ml/min/1.73m^2^)	52(23)	53 (24)	0.59
Ser. creatinine end follow up (mg/dl)	1.43 (0.64)	1.35 (0.68)	0.16
Estimated GFR at end of follow up (ml/min/1.73m^2^)	49 (22)	52 (27)	0.11
24-hour proteinuria 1^st^ year (mg/day)	154 (140)	142.5 (139.5)	0.20
24-hour proteinuria end follow up (mg/day)	157 (212)	126(154)	0.07
Acute rejection, (ever)	11/89 (12.35)	23/187 (12.3)	0.99
Cumulative graft loss 10 years after KTx	6 (6.1)	9 (4.5)	0.55
Cumulative graft loss 15 years after KTx	10 (10.1)	18 (9.1)	0.76
Cumulative graft loss end follow-up	11 (11.1)	18/196 (9.2)	0.56
Graft loss due to primary disease recurrence	2 (2.0)	-	0.5
Graft loss due to other causes	1(1.0)		
Death (from any cause with functioning graft)	3/88 (3.4)	21/183 (11.5)	0.03
Alive with functioning graft (all)	85 (85.9)	161/195 (82.5)	0.98
Alive with functioning graft (after exclusions*)	70/83 (84.3)	82/108 (75.9)	0.15
Follow-up (months)	92.7 (104)	95.7 (110)	0.42
Primary disease recurrence in the graft	23/99 (23.2)	-	-

Abbreviations: IgAN; IgA nephropathy, GFR; glomerular filtration rate, ESKD; end-stage kidney disease.

*Exclusions: ABO incompatible KTx, re-transplant, non-compliance issues.

**Table 3:** Comparison of outcomes between KTx recipients with IgAN as primary cause of ESKD and those with other types of glomerular diseases.

Characteristic	IgAN-KTx	LN-KTx	PIGN-KTx	IMN-KTx	FSGS-KTx
Mean (±sd), median(IQR) or N (%)	Ν = 99	N = 22	N = 21	N = 19	N = 44
Ser. creatinine 1^st^ year (mg/dl)	1.51(0.52)	1.42 (0.39) p = 0.44	1.45 (0.36) p = 0.61	1.58 (0.65) p = 0.60	1.73 (0.80) p = 0.05
Estimated GFR at 1^st^ year (ml/min/1.73 m^2^)	56.0(17.6)	56.5 (16.9) p = 0.71	54.15 (18.7) p = 0.66	48.9 (18.9) p = 0.11	49.9 (11.9) p = 0.0038
Ser. creatinine end fup (mg/dl)	1.55 (0.49)	1.48 (0.3) p = 0.52	1.74 (0.99) p = 0.19	1.53 (0.76) p = 0.88	1.61 (0.7) p = 0.59
Estimated GFR at end of fup (ml/min)	50.5 (15.4)	56.5 (9.9) p = 0.08	46.8 (11.4) p = 0.30	53.1 (20.9) p = 0.52	51.6 (20.9) p = 0.61
24h proteinuria 1^st^ year (mg/day)	154(140)	199.5 (187.5) p = 0.54	110 (93) p = 0.054	209 (600) p = 0.07	585.5 (2875) p = 0.0008
24h proteinuria end fup (mg/day)	157 (212)	60.5 (442) p = 0.18	275(455) p = 0.12	184(243) p = 0.47	1250 (5608) p = 0.0001
Primary disease recurrence (graft)	23 (23.2)	0 p = 0.01	0 p = 0.01	8 (42.1) p = 0.17	26 (59.1)) p<0.0001
Acute rejection, (ever)	11 (11.1)	1 (4.5) p = 0.35	1 (4.7) p = 0.33	3 (9.5) p = 0.83	3 (6.8) p = 0.42
Cumulative graft loss end fup	11 (11.1)	3 (13.6) p = 0.74	2 (9.5) p = 0.81	3 (14.2) p = 0.77	9 (20.45) p = 0.13
Graft loss due to primary disease recurrence	2 (2.0)	0 p = 0.55	0 p = 0.53	0 p = 0.5	5 (11.4) p = 0.0165
Alive with functioning graft	85 (85.9)	18 (81.8) p = 0.62	17 (80.95) p = 0.56	18 (85.7) p = 0.98	34 (77.2) p = 0.2
Follow-up (months)	103.4 (60.9)	80.2 (56.7) p = 0.087	81.2 (50.1) p = 0.1	72.9 (38.7) p = 0.032	64.95 (50.7) p = 0.0003

Abbreviations: IgAN; IgA nephropathy, FSGS; focal segmental glomerulosclerosis, IMN; idiopathic membranous nephropathy, LN; lupus nephropathy, PIGN; pauci-immune glomerulonephritis, ESKD; end-stage kidney disease, GFR; glomerular filtration rate.

Comparisons between the IgAN-KTx group versus all other groups of KTx recipients is depicted by presenting the p-value (p) in each category separately.

#### Graft survival

**Fig 2:**
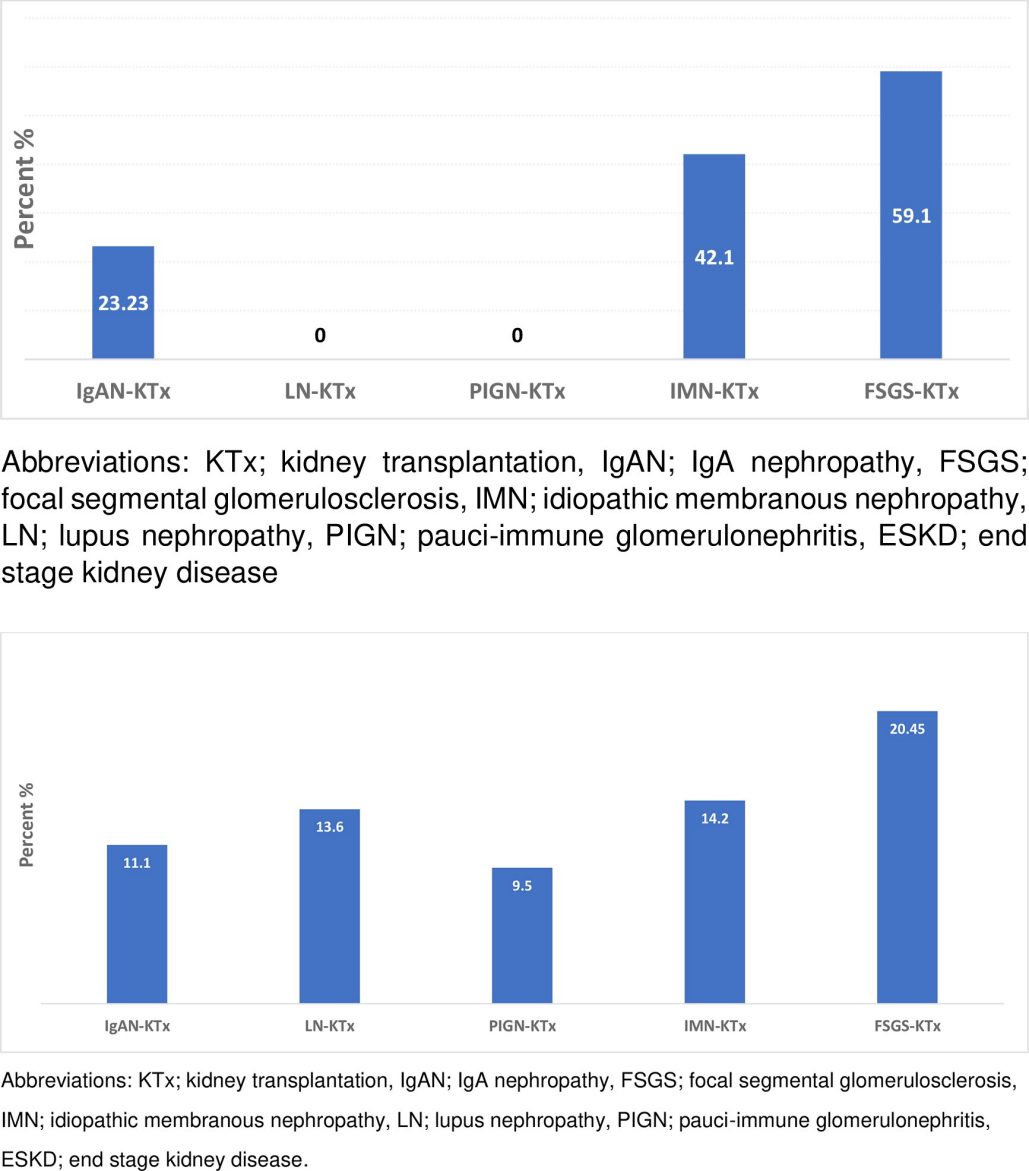
Frequency of primary disease recurrence in the graft among patients with glomerular diseases as cause of ESKD.

Death-censored graft survival was similar in IgAN patients, non-glomerular disease controls and patients with other types of glomerular diseases. However, among recipients with recurrence of primary disease in the graft, patients with ESKD due to IMN, LN, or PIGN were shown to have better graft survival than those with a history of FSGS (Table 3). Among recipients with IgAN, 11.1% of grafts failed, a rate which was not different from the one recorded in the control groups (non-glomerular disease, LN, PIGN, and IMN), but lower than the one observed in the FSGS control group (p = 0.01) (Fig 2). Graft loss due to disease recurrence in the graft was statistically higher in patients with FSGS, compared to all other groups (Table 2).

#### Mortality

Three patients (3.4%) died with a functioning graft in the IgAN group, after a mean time of 67.5(±43.45) months. The cause of death was attributed to malignancies in two of them, and a cardiovascular event in the third one. The cumulative incidence of death in the non-glomerular disease group was higher (11.5%) compared to patients with IgAN (p = 0.03), with the vast majority of deaths attributed to cardiovascular events. The probability of being alive with a functioning graft was similar between groups and remained not different after exclusion of patients with high risk for rejection (Table 3). Within the IgAN group, death-censored graft survival was comparable between patients transplanted from deceased or living donors (90.2% and 87.9%, respectively, p = 0.72).

### IgAN recurrence in the graft

**Fig 3:**
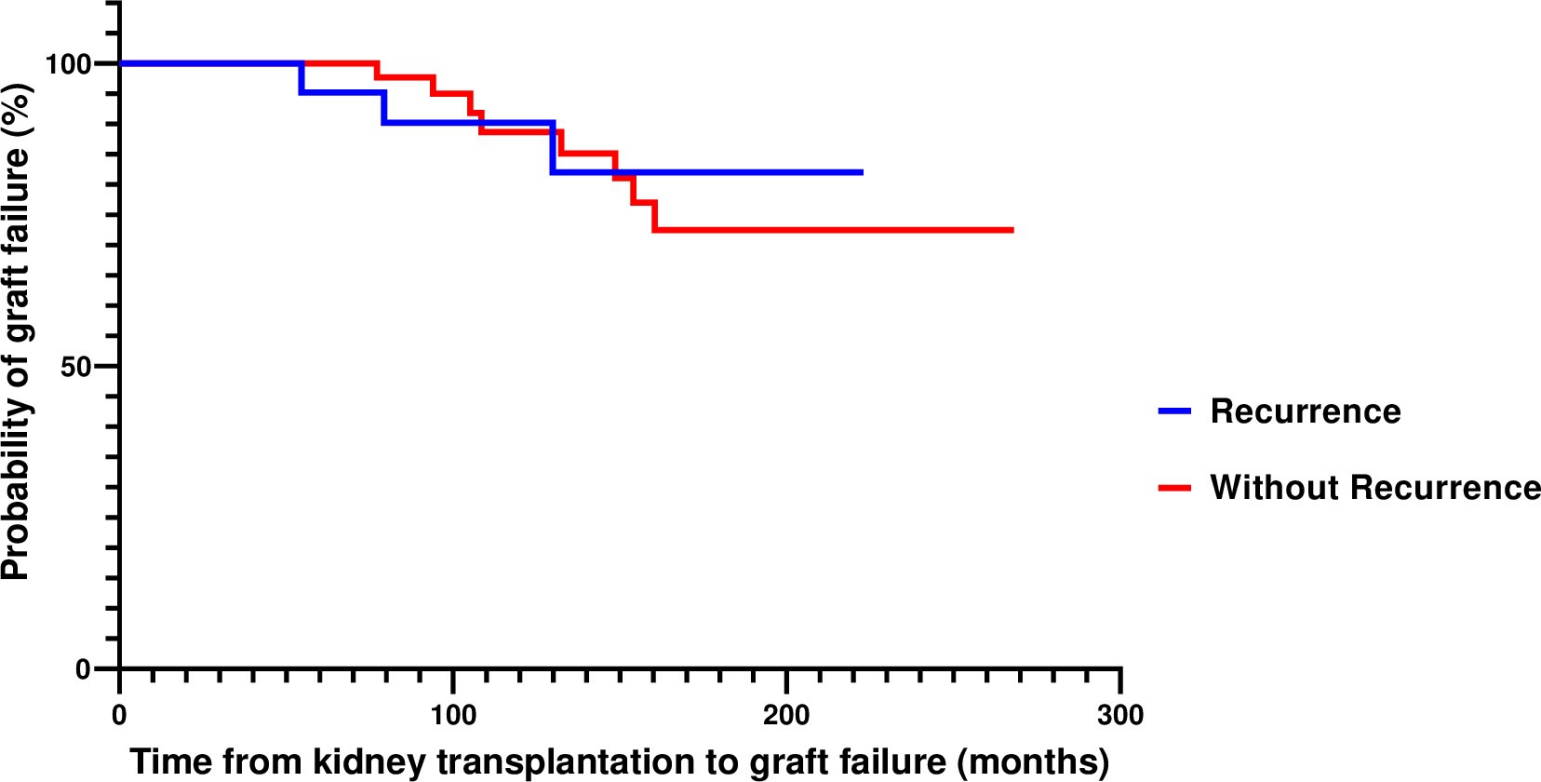
Kaplan Meier estimates of death-censored graft survival in patients with IgAN recurrence in the graft and those without recurrence.

Fifty-two (52.5%) patients with IgAN underwent a graft biopsy, while in 12 of them at least one more biopsy was performed during the study period (Table 4). In 27 cases (51.9%) the indication was related to serum creatinine increases, with or without hematuria or proteinuria. Persistent microscopic hematuria of glomerular origin, with or without proteinuria, was the indication for a graft biopsy in 25 cases (48%). Recurrence of IgAN in the graft was documented in 23 (23.2%) patients. The median time to recurrence was 33 months (range: 7–156). For patients with IgAN recurrence in the graft, the median time from IgAN diagnosis in their native kidneys to renal failure was 3.85 years, and the mean age at IgAN diagnosis in the native kidneys was 35.1(±11.3) years while 73.9% of them were males (Table 4). IgAN recurrence in the graft occurred in 5 (11.9%) out of 41 grafts from deceased donors, and in 18 (31.0%) grafts from living donors (p = 0.027). The mean human leukocyte antigen mismatch score in patients with recurrence was significantly lower, compared to those without recurrence (p = 0.002). The mean serum creatinine at recurrence was 2.1(±0.55) mg/dl, corresponding to an estimated glomerular filtration rate of 42.9(±14.4) ml/min/1.73m^2^, and a median 24-hour proteinuria of 262.5 mg (range: 120–1042). The majority of patients with recurrence were already treated with angiotensin-converting enzyme inhibitors or angiotensin receptor II blockers for arterial hypertension or were started soon after the diagnosis of recurrence. Two patients received immunosuppressive therapy for IgAN recurrence, including a 6-month course of oral glucocorticoids (prednisolone, 1mg per kg of body weight) and intravenous pulses of cyclophosphamide combined with glucocorticoids (one patient). Overall, three patients with IgAN recurrence lost their grafts but only 2 graft failures were attributed to IgAN recurrence (Fig 3). The frequency of living donation was not different between patients with or without recurrence, but living-related donors were significantly more common among patients with IgAN recurrence, versus those without recurrence (80.9% vs. 49.2%, p = 0.01). According to our results, KTx recipients maintained on a regimen including cyclosporine were more likely to experience recurrence of IgAN in the graft, compared to recipients maintained on a regimen containing tacrolimus. Both agents were given in combination with mycophenolate mofetil formulations and low dose methylprednisolone. Within the IgAN group, 9 (39.1%) of patients with recurrence were on cyclosporine at the time of recurrence, and 14 (19.1%) were on tacrolimus (p = 0.02). Yet, graft function was shown significantly better if the patients were treated with tacrolimus [(1.93 (1.2) md/gl versus 1.45 (0.7) mg/dl, p = 0.0019]. Kidney transplant recipients with renal failure due to FSGS experienced disease recurrence in the graft more frequently, compared to patients with IgAN (p<0.0001) and all other groups. Recipients with a history of primary FSGS experienced recurrence early post-transplant, while it was associated with graft failure more frequently than patients with IgAN.

**Table 4:** Comparison of baseline characteristics and outcomes of KTx recipients with IgAN recurrence in the graft versus recipients without IgAN recurrence.

Parameter	Recipients with IgAN recurrence	Recipients without IgAN recurrence	p-value
N (%), Mean (±SD) or Median (IQR)	N = 23	N = 73	
Age at IgAN diagnosis (years)	35.1(10.85)	33.8 (6.7)	0.49
Age at KTx (years)	40.9 (9.6)	44.15 (10.4)	0.18
Gender (male)	17 (73.9)	51 (69.9)	0.71
Time from IgAN diagnosis to ESKD (years)	3.85 (3.3)	5.97 (7.7)	0.13
Dialysis duration (months)	13.06 (34.6)	39.46 (56.1)	0.019
Deceased donor	5 (21.7)	34 (46.6)	0.035
Living donor	18 (78.3)	39 (53.0)	0.027
Living (related) donor	17 (73.9)	25 (64.1)	0.46
Donor age (years)	50.2 (6.1)	53.3 (7.8)	0.11
Serum creatinine at recurrence	2.1(0.8)	-	
Serum creatinine end follow up	1.83 (0.52)	1.44 (0.45)	0.0017
Estimated GFR at end of follow up (ml/min/1.73 m^2^)	42.05 (15.6)	53.9 (14.2)	0.0022
Acute rejection (ever)	2 (9.5)	8 (10.95)	0.84
Graft loss (any cause)	3 (13.0)	8 (10.95)	0.78
Death with functioning graft, any cause	0	4 (5.4)	0.32
Human leukocyte antigen B35	10 (47.6)	21 (29.2)	0.11
Human leukocyte antigen B8	1 (3.0)	4 (5.4)	0.88
Human leukocyte antigen mismatch (all)	1.9 (0.9)	2.4 (0.9)	0.0385
MMF + Tac + GCs	11 (47.8)	54 (73.9)	0.02
MMF + CsA + GCs	9 (39.1)	11 (15.05)	0.01
mTOR + Tac + GCs	3 (13)	8 (10.95)	0.78
Follow up time (months)	121.4 (49.25)	97.4 (65.1)	0.99

Abbreviations: IgAN; IgA nephropathy, GFR; glomerular filtration rate, ESKD; end-stage kidney disease, MMF; Mycophenolate mofetil formulation, Tac; tacrolimus, CsA; cyclosporine, GCs; glucocorticoids, mTOR; mammalian target of rapamycin inhibitor.

#### Histopathology

Appliance of the Oxford criteria for IgAN in the graft biopsies [10] of patients with recurrence revealed that 15 of them (65.2%) had mesangial hypercellularity in >50% of the glomeruli, 9 (39.1%) had segmental glomerulosclerosis, 9 (39.1%) had endocapillary hypercellularity, and 10 (43.8%) had tubular/interstitial fibrosis in >25% of specimen. Crescent formation was found in one patient (4.3%), in 16.7% of the examined glomeruli. Two patients exhibited histological changes consistent with calcineurin inhibitor toxicity, while there was one case with acute cellular rejection and polyoma virus nephropathy documented in the same specimen.

## Discussion

This is a retrospective study comparing outcomes of KTx in 99 consecutive recipients, who ended up in ESKD due to IgAN, with a matched control group of 198 recipients with non-glomerular primary diseases, and four other groups of recipients with glomerular primary diseases, i.e idiopathic FSGS, IMN, LN, and PIGN. All transplants were performed after 2000 in the same center and all study groups were similar regarding the baseline characteristics, except the time in dialysis prior to KTx, which was shorter in the IgAN group, and the frequency of male gender, which was minor in lupus recipients, compared to all others. Long-term graft function and survival in patients with IgAN were similar to all other control groups. However, patients with FSGS recurrence in the graft had a significantly higher probability of graft loss, compared to recipients with recurrent IgAN. Although 23% of IgAN patients experienced disease recurrence in the graft, it was not associated with graft failure but only in 2% of them. The frequency of disease recurrence in the graft was higher in recipients with FSGS versus those with LN or PIGN but not versus patients with IMN as primary disease.

Some studies have reported a more favorable graft survival in IgAN recipients compared to other recipients [11, 12], while others have shown similar results between IgAN patients and controls [13–16]. Most studies, referred to a 5-year follow up time, while reports with longer follow up periods, i.e ten years or longer, have showed that graft survival of IgAN patients was comparable to that of controls [17, 18]. In agreement with our findings, the United Network for Organ Sharing and the Organ Procurement and Transplantation Network database reported that in patients transplanted between 1999–2008, the adjusted hazard ratio for death-censored graft survival was not different between IgAN and controls [18]. Data from the European Renal Association and European Dialysis and Transplant Association [19] registry reported that the risk of death-adjusted graft loss for IgAN recipients was not different from the one observed in patients with polycystic kidney disease until ten years after KTx [19].

The reported rate of IgAN recurrence in the graft in this study lies within the reported range (12.5–35%) across studies following similar methodology with respect to graft biopsy policy [3, 7, 18, 20–24]. However, the range becomes broader if studies using protocol biopsies are taken into consideration [25–27]. Racial and geographical variances in the distribution of IgAN may contribute to these differences, while lack of histological diagnosis of the primary disease or insufficient follow up results in underestimation of the recurrence rate. As shown by the Australia and New Zealand Dialysis and Transplant Registry [20], IgAN recurrence in the graft is a time dependent event, found in a rate of 5.4% at five years and 10.8% at ten years. It is associated with adverse outcomes [3, 4, 28, 29] with the reported rates of graft loss varying substantially between 1.3–17% [18, 25]. Most studies contacted before 2000 reported a rate of graft loss due to recurrence between 3–6% after five years of observation [21, 22, 30, 31], while with longer duration of follow up it was increased to 11% [15]. In our IgAN-KTx cohort, graft loss due to recurrence was uncommon. Potential explanations for these improved results include more efficacious immunosuppressive regimens used for KTx during the last decades [7], which follow a multi-target approach and have impacted the course of IgAN in the graft. All patients received a mycophenolate mofetil formulation, combined with a calcineurin inhibitor, most frequently tacrolimus. Data regarding the efficacy of mycophenolate in IgAN is mixed and thus, current clinical guidelines recommend against its use in IgAN [6, 32]. However, it remains possible that there is a race-specific variability in response to mycophenolate mofetil, or its inclusion in a multi-targeted regimen might be more efficacious, as in LN [33]. Yet, according to our findings, tacrolimus was associated with a lower rate of recurrence within the IgAN KTx population. It has been suggested that serum Gd-IgA1 is predictive of IgAN recurrence in the graft [34], while it has been shown that the levels of serum Gd-IgA1 differ under various immunosuppressants used after KTx [35]. Specifically, it was found, that during the first six post-transplant months, the area under the receiver operating characteristic curve of prednisone was significantly associated with the decrease of Gd-IgA1 and IgA1, whereas area under the receiver operating characteristic curve of tacrolimus was associated with the decrease of IgA1 in the same period. A meta-analysis revealed that tacrolimus combined with low-dose glucocorticoids is an effective and safe therapeutic option for the treatment of IgAN in native kidneys as well [36]. Exploitation of tacrolimus in patients with refractory IgAN has also been tried with promising results [37]. In addition to its immunological effect, tacrolimus may induce proteinuria remission through its hemodynamic effect and the impact on synaptopodin, which results in stabilization of the podocyte cytoskeleton [37].

Recurrence of IgAN after KTx was more frequent in patients who had experienced a shorter disease course prior to ESKD, as a reflection of the various pathogenetic backgrounds across patients with IgAN. A rapidly progressive course of IgAN prior to KTx has been associated with a higher risk of recurrence after KTx in several studies [17, 18, 25, 38, 39]. Yet, younger age at onset of IgAN and greater proportion of crescents on native biopsy predicted recurrence [40] post-transplant. Living donation was associated with a higher probability of recurrence in the present study, while the risk was even higher in transplants from living-related donors, compared to those from living-unrelated donors. This difference had been reported before [17, 22, 28, 38, 41, 42], although disputed by others [15, 18, 24, 32]. Data from the Australia-New Zealand registry reported that IgAN recurrence was more frequent in the lowest human leukocyte antigen mismatching scores in case of living donation [43], a finding, which was also observed in our study.

Patient survival was better in patients with IgAN compared to non-glomerular disease controls, but not different from patients with glomerular diseases, as cause of ESKD. In general, patient survival of KTx recipients with IgAN seems similar or even superior with that of recipients with other primary diseases, as revealed by both registry analyses and single-center studies [4]. Among 32,131 KTx recipients with glomerular primary diseases in the United States in the period 1996–2011, patients with IgAN had the lowest mortality rates, when compared with recipients with renal failure due to other glomerular-diseases or polycystic kidney disease [44]. A registry analysis from United Kingdom reported an unadjusted 10-year patient survival of 85.6% for IgAN recipients and 80.7% for recipients with polycystic kidney disease [45]. Notably, the vast majority of deaths in our non-glomerular disease controls was attributed to cardiovascular events, perhaps as a result of being on dialysis for a longer period.

Limitations of this study pertain to its retrospective design and the relatively small number of patients included in the glomerular-disease groups. However, the frequency of diseases, such as PIGN is relatively low in the general population, while those who end up in ESKD and are eligible for KTx is even smaller. Still, the lack of protocol biopsies precluded the estimation of potential disease recurrence in patients without any evident clinical signs of recurrence.

In conclusion, in our experience, during the past two decades, KTx recipients with IgAN as primary disease, experienced graft outcomes comparable to those of recipients with other causes of ESKD. There was a considerable rate of IgAN recurrence in the graft, which however did not impact graft survival. Of note, KTx recipients who were maintained with a regimen containing tacrolimus had a lower probability to experience IgAN recurrence in the graft, while these patients had better graft function at end, compared to those maintained with a regimen containing cyclosporine [34–37]. In contrast, patients with a shorter course of IgAN prior to ESKD, those who received grafts from living-related donors, or a had lower mismatch score with the donor, had a higher probability of IgAN recurrence in the graft.

## Supporting information

S1 Table Database of kidney transplant recipients with IgA as primary disease. (XLSX)
